# Evaluating ion exchange resin efficiency and oxidative capacity for the separation of uranium(IV) and uranium(VI)

**DOI:** 10.1186/1467-4866-14-1

**Published:** 2013-01-31

**Authors:** Deborah L Stoliker, Nazila Kaviani, Douglas B Kent, James A Davis

**Affiliations:** 1 U.S. Geological Survey, 345 Middlefield Rd, Menlo Park, CA, 94025, USA; 2 Lawrence Berkeley National Laboratory, 1 Cyclotron Rd, Berkeley, CA, 94720, USA

**Keywords:** Anion exchange, Resin separation, Uranium(IV), Uranium(VI), Uranium speciation

## Abstract

**Background:**

Previously described methods to separate dissolved U(IV) from dissolved U(VI) under acidic anoxic conditions prior to laboratory analysis were ineffective with materials currently available commercially. Three strong anion exchange resins were examined for their efficiency in separating, recovering, and preserving both redox states during separation.

**Results:**

Under oxic conditions, recovery of U(VI) from three exchange resins (Bio-Rad AG® 1x8 Poly-Prep® prefilled columns, Bio-Rad AG® 1x8 powder, and Dowex® 1x8 powder) ranged from 72% to 100% depending on the dosed mass, eluent volume, and resin selected. Dowex® 1x8 resin was the only resin found to provide 100% recovery of U(VI) with fewer than 5 bed volumes of eluent. Under anoxic conditions, all three resins oxidized U(IV) in aqueous solutions with relatively low U(IV) concentrations (<3x10^-6^ M). Resin-induced oxidation was observed visually using a leuco dye, safranin-o. Oxidants associated with the resin were irreversibly reduced by the addition of Ti(III). After anoxic resin pre-treatment, a series of U(IV)/U(VI) mixtures at micro-molar levels were prepared and separated using the Dowex® 1x8 resin with 100% recovery of both U(IV) and U(VI) with no resin-induced changes in oxidation state.

**Conclusions:**

Currently available anion exchange resins with apparently identical physical properties were found to have significantly different recoveries for hexavalent uranium at micro-molar concentrations. A novel qualitative technique was developed to visually assess oxidative capacities of anion exchange resins under acidic anoxic conditions. A protocol was developed for pre-treatment and use of currently available anion exchange resins to achieve quantitative separation of U(IV) and U(VI) in aqueous solutions with low U(IV) concentrations. This method can be applied to future work to quantitatively assess dissolved U(IV) and U(VI) concentrations in both laboratory and field samples.

## Background

Uranium exists naturally in the environment within host rocks, soils, groundwaters, and surface waters. Mobilization of naturally occurring uranium can lead to groundwater concentrations in excess of the drinking water standard of 1.3×10^-7^ M or 30 ppb [1,2]. More commonly, mining and processing of uranium ore, as well as nuclear weapons development, has resulted in the development of persistent groundwater plumes with elevated uranium concentrations at a number of sites around the world [3-5]. Under oxic conditions uranium is present as hexavalent U(VI), while under sub-oxic and reducing conditions tetravalent U(IV) is the predominant form [6]. Remediation strategies for many uranium contaminated sites are currently focused on both biotic and abiotic reduction of the readily mobile U(VI) to U(IV) as uraninite, which has a very low solubility at near-neutral and alkaline pH values [7-13].

Recent work has suggested the presence of U(IV) in forms that are potentially more labile than uraninite in uranium-contaminated aquifer and vadose-zone sediments, often attributed to an association with organic matter [14-22]. These non-uraninite-U(IV) phases can be solubilized under aerobic and anaerobic conditions [19,21] however detection of dissolved U(IV) with laboratory-based (non-synchrotron) instrumentation is not possible due to the rapid oxidation of U(IV) to U(VI) under conditions typically required for instrumental determinations of low concentrations of uranium. Kinetic phosphorescence analysis (KPA) offers a means to detect only dissolved U(VI) due to the luminescent nature of hexavalent uranium and non-luminescence of other valence states [23,24]. However, oxidation of U(IV) may occur during sample exposure to atmospheric conditions or acidification with nitric acid, commonly required for analysis.

For this reason, separation of dissolved U(IV) and U(VI) under anaerobic conditions prior to contact with nitric acid and instrumental analysis, is desired. Strongly basic anion exchange resins have been used previously to separate U(IV) and U(VI) [7,25-28]. These resins are composed of quaternary trialkylammonium functional groups attached to a styrene divinylbenzene polymer lattice [29,30]. In hydrochloric acid solutions of concentrations greater than 3.5 M, U(VI) forms anionic chloro-complexes which adsorb extensively to the resin. In contrast, U(IV) remains as cationic species which adsorb minimally below HCl concentrations of 5.5 M [25,27,28]. Separation of the two oxidation states can be achieved by introducing a 4 M HCl solution containing U(IV) and/or U(VI) to a column packed with anion exchange resin, flushing with 4 M HCl, and collecting the eluate. Under these conditions, only U(IV) will pass through the resin bed. Uranium(VI) is then eluted with 0.1 M HCl, which allows the U(VI)-chloro complexes to dissociate. Analysis of the separate fractions can then be carried out via KPA after sample preparation during which U(IV) fractions will be oxidized. In order to achieve uranium mass balance and quantify dissolved concentrations, both U(IV) and U(VI) recoveries need to be reproducible and complete (i.e. 100%). In addition, any functional groups or constituents associated with the resin must not oxidize U(IV) nor reduce U(VI). Both of these issues, poor recovery and resin-induced U(IV) oxidation, were observed by the authors when first attempting to utilize currently available commercial anion exchange resins for the separation of U(IV) and U(VI) even in simple, aqueous hydrochloric acid solutions. Many recent studies have focused on the use of anion-exchange resins to remove U(VI) from natural systems under ambient conditions [30-33]. However, to our knowledge, the issue of resin-induced oxidation of U(IV) under the acidic conditions necessary for U(IV)/U(VI) separation has not been reported. In this paper, we evaluate the use of anion exchange resins currently available commercially for U(IV)/U(VI) separation, present novel techniques to provide immediate visual confirmation of whether anion exchange resins possess oxidative capacity, and develop a protocol that can be used to achieve quantitative separation of U(IV) and U(VI) from anoxic hydrochloric acid solutions with micro-molar uranium concentrations. Complications associated with potential oxidation of U(IV) by constituents such as Fe(III) present in environmental samples are eliminated by working in simple, aqueous hydrochloric acid solutions [6].

## Methods

### Recovery of U(VI) under oxic conditions

**Figure 1:**
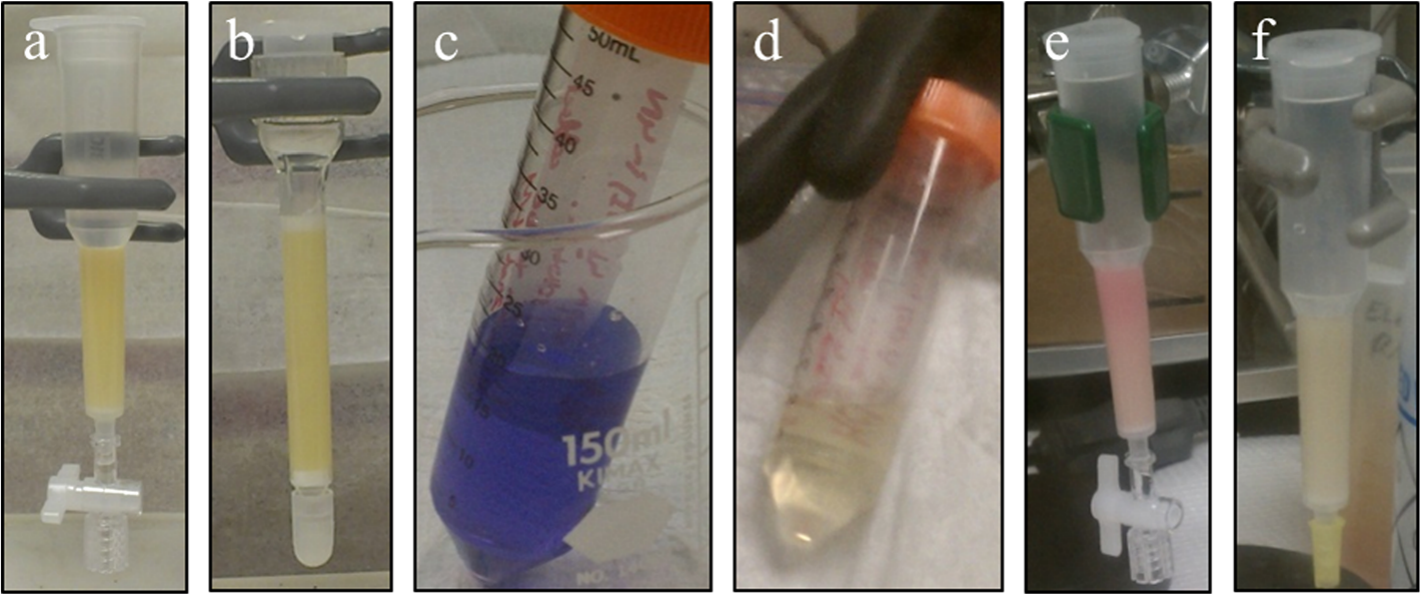
**Use of safranin-o leuco dye as a visual oxidation indicator. a**) Bio-Rad® AG1x8 resin in Poly-prep column including a stop-cock valve to control flow without indicator dye showing original color of the resin; **b**) Dowex® 1x8 resin in Pierce polystyrene column including top and bottom frits to control flow without indicator dye showing original color of the resin; **c**) Solution of safranin-o in the oxidized form (1 drop of 1% solution in 4 M HCl); **d**) Anoxic safranin-o (colorless, reduced form) in 4 N HCl after reduction with Ti(III); **e**) Example of anoxic resin (no pre-treatment) after addition of reduced safranin-o solution with visible oxidation (pink color on resin); **f**) Anoxic resin after pre-treatment with Ti(III) and addition of reduced safranin-o solution (no color change observed).

Before evaluating U(IV)/U(VI) separation and recovery, the efficiency of U(VI) recovery using several different resins was examined under oxic conditions. Based on cost, descriptions in published studies, and previous experience in our laboratory, three strong anion exchange resins in the chloride form (100-200 mesh with 1.2 meq /mL exchange capacity) were selected for testing: Bio-Rad AG® 1×8 Poly-Prep® prefilled columns, Bio-Rad AG® 1×8 powder, and Dowex® 1×8 powder. The use of trade, product, or firm names herein is for descriptive purposes only and does not imply endorsement by the U.S. Government. Resins were packed and evaluated in various columns, including both the Bio-Rad Poly-Prep® polypropylene columns (0.8 cm ID with a Kynar® 20 μm frit resin bed support and an attached luer-slip, 2-way stop-cock) and Thermo Scientific® Pierce® polystyrene columns (0.7 cm ID with two 30 μm polyethylene discs situated above and below the resin bed) (Figure 1). Bed volumes calculated for packed columns were 1.4-2 mL (corresponding to exchange capacities of 1.7-2.4 meq). Powder resins were pre-washed in batch prior to column packing by suspension in 0.1 M HCl, settling, and decanting the supernatant. This step was performed with ~2-5 g of resin mixed with ~20 mL of acid four times. A final addition of 20 mL of 0.1 M HCl was used to slurry pack the column. Resin columns were then flushed with at least 10 bed volumes of 4 M HCl prior to addition of uranium-containing solutions (Table 1). Pre-packed resins were pre-washed by draining the packing solution (deionized water), flushing with 3 bed volumes of deionized water with a resistivity of >18 MΩ^.^cm followed by 0.1 M HCl, and flushing with at least 10 bed volumes of 4 M HCl. All hydrochloric acid solutions were prepared from dilution of trace-metal grade concentrated acid (Thermo Fisher Scientific, Waltham, MA). Oxic uranium secondary stock solutions (2×10^-6^ M, 8×10^-6^ M, and 2×10^-5^ M) were prepared by dilution of a primary stock in 4 M HCl. The primary uranium stock solution (1×10^-2^ M) was prepared by dissolution of UO_3_ in hydrochloric acid. Secondary uranium stock solutions (1 mL) were added to the resin-packed columns, eluted with multiple bed volumes (3-6) of 4 M HCl collected as separate fractions of ~2 mL, and then eluted with multiple bed volumes (6-10) of 0.1 M HCl collected as separate fractions of ~2 mL.

**Table 1:** Adopted method for the anoxic separation of U(IV)/U(VI) at micro-molar concentrations

**Step**	**Protocol (in anoxic glovebag with anoxic solutions)**	**Purpose/Importance**
1	Wash ~2-5 g of Dowex® 1x8 resin (100–200 mesh, chloride form) with ~20 mL 0.1 M HCl, repeat 4x	Pre-wash to remove any residual material from resin production that can interfere with uranium analysis via KPA
2	Use final rinse from Step 1 to slurry-pack a suitable column (such as a Thermo Scientific® Pierce polystyrene column, 0.7 cm ID with top and bottom frits) to 1.5-2 mL bed volume	Use a bed-height to column diameter ratio >3. In this study, bed-height to column diameter ratios between 5 and 7 were used with success
3	Flush resin-packed column with 4 M HCl (~10 bed volumes)	Pre-flush to ensure resin column is completely saturated with 4 M HCl
4	Add two successive flushes of 1x10^-2^ M Ti(III) (2 bed volumes each) in 4 M HCl and, using end caps, seal in a final 1.5-2 bed volumes of Ti(III) and allow the packed resin column to soak for 3 or more days	Pre-treat resin to reduce oxidants associated with the resin that cause oxidation of U(IV)
5	Drain the resin column and flush with at least 10 bed volumes of 4 M HCl	To flush out residual Ti(III) prior to sample addition
6	Add uranium-containing solution in 4 M HCl (1 mL). Elute column with a total of 9 bed volumes of 4 M HCl and collect fraction(s)	To collect U(IV) fractions, which pass through the resin at 4 M HCl
7	Elute column with a total of 9 bed volumes of 0.1 M HCl and collect fraction(s)	To collect U(VI) fractions, which adsorb to resin at 4 M HCl but are eluted at 0.1 M HCl
8	Flush column with a total of 7 bed volumes of 0.1 M HCl	Post-flush to ensure all U(VI) is flushed away and clean resin for future use
9	Flush column with a total of 7 bed volumes of 4 M HCl	Post-flush to ensure all U(IV) is flushed away and resin column is in 4 M HCl state for future use
Column is ready to be re-used by repeating Steps 6–9 (Steps 1–5 need only be completed once)		

A split of each fraction was dried in a glass scintillation vial on a hot plate and converted to a nitrate salt by repeated drying after addition of 1 mL of concentrated nitric acid (Thermo Fisher Scientific, Waltham, MA) and 0.2 mL of 30% hydrogen peroxide (Thermo Fisher Scientific, Waltham, MA). These steps are necessary to avoid KPA interferences caused by Cl^-^ and dissolved organic matter. Fractions for column separations and all other samples analyzed to determine uranium concentrations were treated similarly.

To determine dissolved U(VI) concentrations, the dried nitrate salts were then reconstituted in 0.15 M nitric acid and analyzed on a Chemchek KPA-11 (Chemchek, Richland, WA). Briefly, the diluted uranium solution was mixed with a proprietary complexing agent that enhances its photoluminescence. The sample was excited by a pulsed laser and the phosphorescent intensity vs. time recorded [24]. For each analysis, 1000 laser pulses were averaged and sample lifetimes ranged between 280 and 300 μs. The analytical range for quantifying uranium concentrations was 1×10^-9^ to 1×10^-6^ M with standard deviations of 3% based on repeated determinations of quality control standards measured over the time period during which experiments were conducted.

### Recovery of U(IV) and U(VI) under anoxic conditions

All anoxic solutions were prepared in an anaerobic chamber (Coy Lab Products, Grass Lake, MI) with an atmosphere of 2-5% hydrogen balanced with nitrogen. Two fan boxes within the glovebag cycled the atmosphere through desiccant filled palladium Stak-Paks to control oxygen and humidity. Solutions were sparged using gas dispersion tubes with the hydrogen-nitrogen gas mix for several hours (depending on the volume of solution) and checked for dissolved oxygen colorimetrically with a CHEMetrics (Midland, VA) vacu-vial filled with Rhodazine D™, with an minimum detection limit of 1.6×10^-6^ M. All laboratory supplies (e.g. pipette tips, sample vials, resin, resin columns, etc.) were equilibrated in the glovebag for a minimum of 24 hours prior to use. U(IV) secondary stock solutions were prepared by adding 10^-3^ M or 10^-2^ M Ti(III) in 4 M HCl to anoxic U(VI) secondary stock solutions [27]. To prevent an excess of reactive Ti(III) in solution, sub-stoichiometric amounts of Ti(III) were added to achieve solutions <100% U(IV) (i.e. partly U(IV) and partly U(VI)). Anoxic Ti(III) solution was obtained from Thermo Fisher Scientific as 20% TiCl_3_ in 2 M HCl purged with N_2_. Neither Ti(III) nor the resulting oxidized form, Ti(IV) produced by reaction with U(VI), sorb to the anion exchange resin in the presence of 4 M HCl or 0.1 M HCl [26]. Resin pre-washing and column packing were carried out in the same manner described for oxic recovery checks inside the glovebag with anoxic solutions. Further treatment steps required for anoxic separation are detailed in the results section.

## Results and discussion

### Recovery of U(VI) under oxic conditions

**Figure 2:**
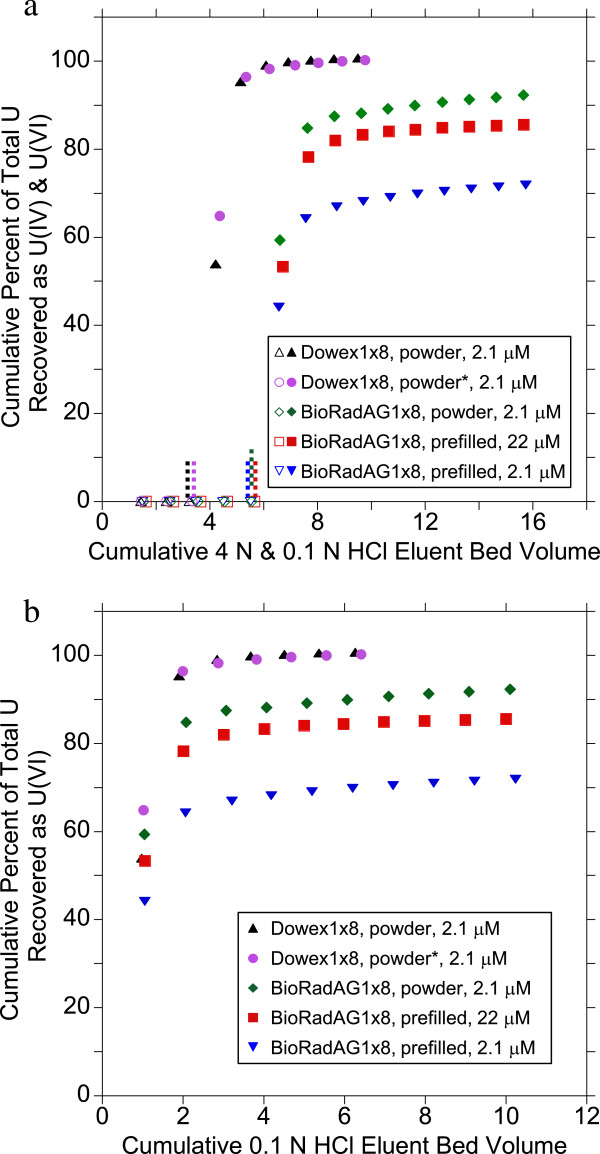
**Recovery of U(VI) from various resins under oxic conditions.** Resin separation of oxic U(VI) solutions using Bio-Rad® AG1x8 prefilled Poly-prep columns, Bio-Rad® AG1x8 powder packed in the Poly-prep columns and Dowex® 1x8 powder packed in the Poly-prep and Pierce polystyrene(*) columns showing a) cumulative recovery of total uranium for both 4 M HCl (open symbols, U(IV)) and 0.1 M HCl (closed symbols, U(VI)) column elution with dashed lines indicating where the eluent composition was changed for each system and b) cumulative recovery of total uranium as U(VI) from only 0.1 M HCl (U(VI)) elutions.

Bio-Rad AG® 1×8 Poly-Prep® prefilled columns were originally selected for U(IV)/U(VI) separation as this resin has been used previously for this task [7,27] and arrives pre-packed in a column ready for use. A two-way stop-cock valve was added to the column to prevent pore fluid from draining completely between eluent additions (Figure 1a). Recovery of U(VI) under oxic conditions was tested for this resin using 2.1×10^-6^ M, and 2.2×10^-5^ M uranium solutions in 4 M HCl. After preparing the resin columns, which included pre-washing, 1 mL of uranium solution was added followed by a minimum of 5 bed volumes (10 mL) of 4 M HCl eluent with measured flow rates of ~0.45 mL/min. No uranium could be detected in any of the 4 M HCl fractions confirming strong adsorption of U(VI) to the resin (Figure 2a). Subsequent elution with 0.1 M HCl resulted in slow recovery of U(VI) (Figure 2). Recovery of U(VI) ranged from 72% for a 2.1×10^-6^ M uranium solution to 86% for a 2.2×10^-5^ M solution after 9 bed volumes of 0.1 M HCl were passed through the column (Table 2). The slow, incomplete, recovery of U(VI) from these pre-packed columns precluded their use in U(IV)/U(VI) systems, where quantification requires 100% recovery with reasonable volumes of eluent. New Poly-Prep® columns were emptied, rinsed, re-packed with a pre-washed slurry of Bio-Rad AG® 1×8 in powder form, 1 mL of the 2.1×10^-6^ M U(VI) solution was added, and the columns eluted with 4 M and 0.1 M HCl (flow rate of ~0.63 mL/min). Uranium(VI) recovery improved over the prefilled columns from 72% to 92% after 10 bed volumes of 0.1 M HCl (Figure 2, Table 2). However, the requirement for 100% recovery of micro-molar concentrations of U with small elution volumes could not be achieved with this resin.

**Table 2:** Summary of selected resin separation tests including treatment steps and outcome

**Resin**	**Column Type**	**Oxic or anoxic conditions**	**Ti(III) treatment**	**Initial [U**_**tot**_**] (μM)**^**a**^	**U**_**tot**_**Recovered (%)**^**a**^	**Initial U(IV) (%)**	**Initial U(VI) (%)**	**Recovered as U(IV) (%)**^**b**^	**Recovered as U(VI) (%)**^**b**^
Bio-Rad AG1x8 prefilled	Bio-Rad Poly-prep	Oxic	No	2.1	72	0	100	0	100
Bio-Rad AG1x8 prefilled	Bio-Rad Poly-prep	Oxic	No	22.2	86	0	100	0	100
Bio-Rad AG1x8 powder	Bio-Rad Poly-prep	Oxic	No	2.1	92	0	100	0	100
Dowex 1x8 powder	Bio-Rad Poly-prep	Oxic	No	2.1	100	0	100	0	100
Dowex 1x8 powder	Pierce polystyrene	Oxic	No	2.1	100	0	100	0	100
Bio-Rad AG1x8 prefilled	Bio-Rad Poly-prep	Anoxic	No	2.1	81	36	64	0	100
Dowex 1x8 powder	Pierce polystyrene	Anoxic	No	8.7	100	98	2	86	14
Dowex 1x8 powder	Pierce polystyrene	Anoxic	Yes	8.7	100	98	2	98	2
Dowex 1x8 powder	Pierce polystyrene	Anoxic	Yes	1.8	100	0	100	0	100
Dowex 1x8 powder	Pierce polystyrene	Anoxic	Yes	2.8	100	100	0	100	0
Dowex 1x8 powder	Pierce polystyrene	Anoxic	Yes	2.1	100	10	90	11	89
Dowex 1x8 powder	Pierce polystyrene	Anoxic	Yes	1.8	100	48	52	47	53

a. 1 mL of uranium solution added to resin, uncertainty in recovered U_tot_ was 3%.

b. Uncertainty in speciation recovery was 1%.

Hussonnois et al. [28] reported the use of Dowex® 1×8 resin for U(IV)/U(VI) separation so this resin was also selected for evaluation. Dowex® 1×8 powder resin was pre-washed, slurry-packed and tested in both the Poly-Prep® columns and Thermo Scientific® Pierce® polystyrene columns. The latter type of columns were packed with frits above and below the resin bed as recommended by the manufacturer making the use of a valve to stop flow unnecessary (Figure 1b). In both types of columns, 100% of the total uranium in a 1 mL addition of 2.1×10^-6^ M U(VI) solution was eluted after only 3 bed volumes (~5.5 mL with a flow rate of ~0.45 mL/min) of 0.1 M HCl eluent had been added (Figure 2, Table 2). Thus, complete recovery of micro-molar uranium concentrations with small eluent volumes was best achieved with this resin and a total of 9 bed volumes of 0.1 M HCl eluent was chosen for the standard protocol (Table 1). A bed-height to column diameter ratio of ~7 was used in testing the Dowex 1x8® resin with 100% recovery (an identical ratio is inherent in the Bio-Rad® prefilled columns). However, when a larger diameter column was packed with Dowex 1x8® resin with similar bed volume (~2 mL) and a bed-height to column ratio of ~3, total recovery decreased to 95%. Based on the 1 σ standard deviation, total recoveries from 25 replicate measurements with Dowex 1×8® were 97-103%. Consequently, the uncertainty in total recovery was assigned a value of 3%. Replicate resin separations revealed highly reproducible results for each individual fraction collected with standard deviations between replicate fractions of 0.4%.

### Recovery of U under anoxic conditions – Oxidation of U(IV) by resin and Ti(III) treatment

Solutions with U(IV) were produced by adding sub-stoichiometric amounts of anoxic Ti(III) to an anoxic U(VI) solution in an anaerobic chamber to produce the desired U(IV)/U(VI) composition. A solution (1 mL) with 8.7×10^-6^ M total uranium concentration containing of 98% U(IV) and 2% U(VI) was passed through an anoxically prepared Dowex® 1x8 resin-packed column. Only 86% of the uranium was recovered as U(IV); the remaining 14% was recovered as U(VI) suggesting resin-induced oxidation of U(IV) (Table 2). Similar oxidation results were obtained with the Bio-Rad AG® 1×8 Poly-Prep® prefilled columns. When 1 mL of a 36% U(IV) and 64% U(VI) solution (2.1×10^-6^ M total uranium concentration) was added to an anoxically prepared column, all of the recovered uranium was U(VI) (81% total recovery). The maximum uncertainty in speciation was 1% based on duplicate recoveries of various U(IV)/U(VI) mixtures and comparison of known and experimentally determined uranium speciation (0/100, 36/64, 98/2, and 100/0, described in subsequent method validation section).

The oxidative capacity of both the Dowex and Bio-Rad resins was estimated to be ~0.002 meq per liter bed volume under anoxic hydrochloric acid conditions. While this low value will not have a measureable impact on solutions with very high tetravalent uranium concentration, the oxidative capacity of a 2 mL bed volume resin column is sufficient to completely oxidize 1 mL of a 1.0×10^-6^ M U(IV) solution (10 times the drinking water limit). Thus the implications for lower-level, field-relevant, concentrations are significant. The nature of the trace-constituent oxidant present on the resins is unknown, but the most likely oxidant is trace amounts of oxygen trapped in the resin. A dissolved oxygen concentration of 0.5×10^-6^ M could account for the observed oxidation of U(IV). Alternatively, the oxidant could be a residual from proprietary manufacturing processes. Redox reactive functional groups, such as nitro moieties, or other known oxidizers of uranium, such as nitrate or Fe(III), could be present at sufficiently low concentrations to make detection of the specific oxidant difficult.

Since the time from sample collection to KPA uranium measurement is a few days with the chloride removal step, a visual redox indicator was added to the system to assess oxidation more rapidly. Safranin-o (C_20_H_19_ClN_4_) is a leuco dye redox indicator whose oxidized form is vibrant purple, red, or pink but is colorless when reduced. One drop of 1% safranin-o in the oxidized form (Thermo Fisher Scientific) was added to a 20 mL uranium solution (8.4×10^-6^ M) in 4 M HCl under oxic conditions resulting in a strong purple color (Figure 1c). This solution was sparged in the glovebag to remove any dissolved oxygen and 1×10^-3^ M Ti(III) was then added drop-wise until the solution turned colorless (Figure 1d). The solution remained colorless under anoxic conditions in the glovebag but developed a purple color, characteristic of the oxidized form, over the course of a few hours once removed from the glovebag and exposed to atmospheric conditions. Under anoxic conditions, the reduced uranium solution with indicator was added to a resin-packed column and an immediate color change to bright pink was observed on the resin (Figure 1e). All three resin types showed a color change characteristic of the oxidized form of the indicator throughout the resin bed. Addition of a 1×10^-3^ M Ti(III) solution (without uranium) to the dyed resin resulted in the conversion of the indicator back to its colorless, reduced, form. Based on these results, a pre-treatment step was added to the resin packing protocol. Following pre-washing and column packing with Dowex® 1×8, three bed volumes of 1×10^-3^ M Ti(III) were flushed through the resin. The column was capped at the bottom, a final 1.5 bed volume aliquot of Ti(III) added, the top sealed with a cap, and the system left to soak overnight. The redox equivalents in this solution are 4 orders of magnitude greater than the amount necessary to reduce the resin-bound oxidants based on the resin oxidative capacity. After the overnight equilibration, the column was flushed with ~12 bed volumes of 4 M HCl to remove any residual titanium. Fractions were collected, diluted with 0.15 M nitric acid, and analyzed for titanium via ICP-OES (detection limit 1e^-7^ M). Complete recovery of titanium was achieved within 4 bed volumes of 4 M HCl eluent. To the pre-treated and flushed column, 1 mL of a reduced uranium solution with safranin-o indicator, in the colorless form, was added. A color change to pink was observed, indicating oxidation of some of the indicator, although it was less pronounced than it had been without Ti(III) treatment. Oxidation of U(IV) was also measured in the eluted fractions. Therefore, a more rigorous procedure was tested. A 1.0×10^-2^ M Ti(III) solution was used in place of the 1.0×10^-3^ M Ti(III) solution to pre-treat the resin. The resin was flushed with three bed volumes, after which the system was capped and allowed to soak for ~3 days prior to flushing with 4 M HCl. We hypothesize that elevated Ti(III) concentrations and contact times are required to reduce all resin-bound oxidants as neither Ti(III) nor Ti(IV) sorb to the anion exchange resin in the presence of 4 M HCl [26]. Reduced indicator solutions added to resins treated in this manner remained colorless, indicating the oxidative capacity of the resin was depleted (Figure 1f). Resin pre-treatment with 1.0x10^-2^ M Ti(III) was selected for the standard protocol (Table 1).

### Recovery of U(IV) under anoxic conditions – Method validation

**Figure 3:**
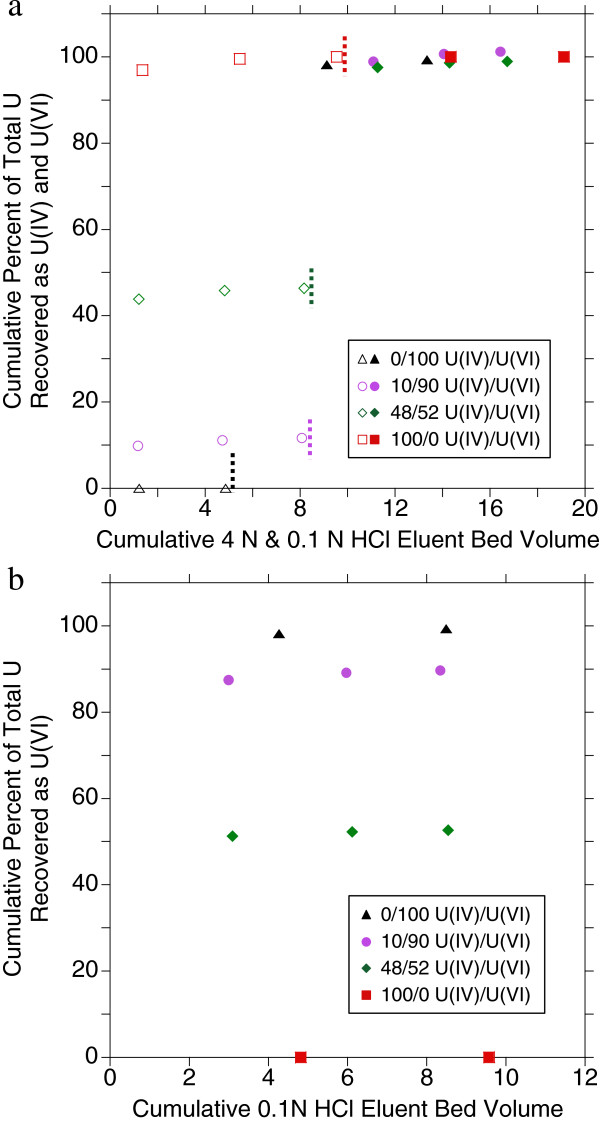
**U recovery from Dowex**® **1x8 resin under anoxic conditions with varied U(IV)/U(VI) solutions.** Dowex® 1x8 resin separation of anoxic solutions consisting of 0%, 10%, 48% and 100% U(IV) after resin pre-treatment showing **a**) cumulative recovery of total uranium for both 4 M HCl (open symbols, U(IV)) and 0.1 M HCl (closed symbols, U(VI)) column elution with dashed lines indicating where the eluent composition was changed for each system and **b**) cumulative recovery of total uranium as U(VI) from only 0.1 M HCl (U(VI)) elutions.

Using the more rigorous reductive pre-treatment protocol, a series of tests was conducted on newly prepared Dowex® 1×8 resin columns without the addition of the leuco dye. First, a 1.8x10^-6^ M U(VI) solution was passed through the resin and eluted with 4 M HCl and 0.1 M HCl to ensure no excess reductants had been deposited on the resin. All uranium was recovered in the hexavalent form (100% total recovery, Figure 3, Table 2). Similar to oxic U(VI) tests, full recovery of U(VI) under anoxic conditions was achieved within 4 bed volumes of 0.1 M HCl eluent and a total of 9 bed volumes was chosen for the standard protocol (Table 1). Next, a pure U(IV) fraction was collected by passing 1 mL of a 8.7×10^-6^ M total uranium concentration (98% U(IV)) through a separately prepared resin-packed column and eluting with 2 mL of 4 M HCl for a final uranium concentration of 2.8×10^-6^ M. This 100% U(IV) solution was passed through a separately prepared resin-packed column. Complete U(IV) recovery was achieved after approximately 5 bed volumes of 4 M HCl elution and a total of 9 bed volumes of 4 M HCl eluent was chosen for the standard protocol (Table 1). Resin separation of the pure U(IV) solution showed 100% total recovery and 100% U(IV) content (Figure 3, Table 2). To determine whether packed columns can be reused, the column used in the previous test was post-flushed with 6 additional bed volumes of 0.1 M HCl and then 6 bed volumes of 4 M HCl and the separation experiment was repeated. Results were identical with 100% total recovery and 100% as U(IV).

Four months later, the same original 98% U(IV) solution was passed through a freshly prepared resin column with 100% total uranium recovery and 92% as U(IV). The minimal amount of U(IV) oxidation over many months suggested by these results indicates the stability of the Ti(III) reduced systems under anoxic conditions. Finally, the first fraction from the previous test, comprised of 1 mL of U(IV) solution and 2 mL of 4 M HCl eluent with a total concentration of 2.6×10^-6^ M, was collected to have a pure U(IV) fraction to admix with U(VI). Both a 48% U(IV) / 52% U(VI) and a 10% U(IV) / 90% U(VI) solution were prepared by mixing an aliquot of the pure U(IV) fraction with an aliquot of anoxic U(VI) solution for final total uranium concentrations of 2.1×10^-6^ M and 1.8×10^-6^ M respectively. When these solutions were passed through pre-washed, pre-treated resins and eluted the total uranium recovery was 100% for each test with measured U(IV)/U(IV) ratios of 47/53 and 11/89 respectively (Figure 3 and Table 2).

## Summary and conclusions

In choosing the proper strong base anion exchange resin for separation of redox sensitive species, careful consideration should be paid not only to the efficiency of recovery of each constituent but also the potential for resin-induced oxidation. Three different resins (two from the same manufacturer) with identical mesh size, base form, and exchange capacity were found to sorbed U(VI) very strongly in 4 M HCl but recoveries of U(VI), from elution with 0.1 M HCl, varied between 72% and 100%. In addition, all three resins were found to cause oxidation of U(IV) under anoxic conditions in a glovebag. Oxidative properties were observed by measuring U(IV) and U(VI) but also by the addition of a leuco dye to the resin, which allowed an immediate visual indication of redox transformations. The use of visual redox indicators, when possible, can offer significant time savings in method development and testing when sample analysis is a lengthy process. The oxidative capacity of these anion exchange resins was eliminated by treatment with the reducing agent Ti(III). To achieve U(IV)/U(VI) separation, soaking resin-packed columns in a solution of 1x10^-2^ M Ti(III) permanently removed the residual oxidants present on the resin. Ti(III) was completely flushed from the column and did not further impact results (e.g. reduction of U(VI) or instrumental interferences) with future resin use. After pre-treatment, U(IV)/U(VI) resin separation was found to be accurate, reproducible and packed resin columns reusable after post-separation flushing. Future work utilizing this treatment technique will be applied to field-collected anoxic sediments for the characterization of associated U(IV) and U(VI).

## Competing interests

The authors declare that they have no competing interests.

## Authors’ contributions

DLS designed and conducted the experiments, prepared/analyzed KPA and ICP samples, interpreted the data, and wrote this manuscript. NK performed the resin prewashing and pretreatment steps, prepared/analyzed KPA samples, conducted the leuco-dye tests and two untreated anoxic resin separations included here along with many additional tests which are not included but significantly impacted the final experimental design. DBK and JAD helped revise this manuscript and offered experimental design suggestions. All authors read and approved the final manuscript.
